# A rapid isothermal RPA–CRISPR/Cas12a assay for detection of *Rickettsia rickettsii*

**DOI:** 10.3389/fmicb.2026.1823193

**Published:** 2026-04-16

**Authors:** Sezayi Ozubek, Huitao Liu, Roman R. Ganta

**Affiliations:** Department of Pathobiology and Integrative Biomedical Sciences, College of Veterinary Medicine, Bond Life Sciences Center, University of Missouri, Columbia, MO, United States

**Keywords:** isothermal amplification, rapid detection, *Rickettsia rickettsii*, Rocky Mountain spotted fever, RPA–Cas12a assay, molecular diagnosis

## Abstract

**Introduction:**

Rocky Mountain spotted fever (RMSF) resulting from the tick-borne *Rickettsia rickettsii* infections is a potentially fatal tick-borne disease affecting humans and dogs in the Americas. It is difficult to confirm the diagnosis early in the laboratory on account of low-level and inconsistent rickettsemia in whole blood.

**Methods:**

Here we established a fast, fully isothermal RPA–CRISPR/Cas12a assay that targeted the vitamin uptake transporter (*vut*) gene of *R. rickettsii*. Two independent crRNA primer sets (104 bp and 92 bp) were developed to independently amplify the gene target to enhance the reliability and specificity of the assay.

**Results:**

Using quantified synthetic DNA (gBlock) standards derived from the *R. rickettsii* Sheila Smith *vut* target region, the assay was able to detect target DNA in about 40 min at 37 °C with an observed analytical detection limit of 60–70 copies per reaction. Specificity testing on four *R. rickettsii* strains and a panel of non-target spotted fever group rickettsiae and tick-borne bacteria of clinical importance demonstrated no cross-reactivity of the assay with these pathogen nucleic acids. Applied to archived longitudinal canine whole-blood DNA extracts from experimentally infected dogs (*n* = 6), the performance was intermittent for known limitations of whole blood testing in RMSF and limited to specific post-infection days.

**Discussion:**

The findings of this study support the feasibility of RPA–Cas12a as a rapid molecular workflow for *R. rickettsii* detection, while indicating that broader validation in additional clinically relevant and removed tick sample types is still needed.

## 1 Introduction

Rocky Mountain spotted fever (RMSF) has been known for over a century but still remains one of the most severe tick-borne diseases in humans and dogs throughout the Americas (Wolbach, 1919; Ganta, 2022; Latré De Laté et al., 2025). It is caused by *Rickettsia rickettsii*, an obligate intracellular bacterium that is transmitted by ticks, and its transmission is strongly influenced by local tick vector ecology (Ganta, 2022). In the United States, *Dermacentor* var*iabilis* and *D. andersoni* are most commonly associated with RMSF transmission, although in portions of Central and South America several *Amblyomma* species have also been reported as vectors (Nicholson et al., 2010; Laukaitis and Macaluso, 2021). Furthermore, the outbreaks linked to the *Rhipicephalus sanguineus* species complex typically in peridomestic environments, such as in the Indian reservations in Arizona and Northern New Mexico of the USA, where dogs are in close proximity with humans serving as a reminder that RMSF is not confined only to the classic endemic maps and can re-emerge when local vector ecology and living conditions facilitate transmission (Álvarez-Hernández et al., 2017; Sánchez Pérez et al., 2023; Brophy et al., 2025; Rubino et al., 2025).

Rocky Mountain spotted fever is an acute and potentially life-threatening disease in both humans and dogs (Ganta, 2022). In both hosts, early clinical signs are non-specific, which can delay recognition and timely treatment (Gottlieb et al., 2018; Álvarez-Hernández et al., 2024). Dogs may experience high fever, lethargy, anorexia, vomiting and thrombocytopenia although more severe clinical disease can include ocular lesions, bleeding tendencies (petechiae/ecchymoses), arthralgia and neurologic signs (Labruna et al., 2009; Kidd and Breitschwerdt, 2013; Levin et al., 2014; Latré De Laté et al., 2025). A key event in pathogenesis is infection of vascular endothelial cells and development of systemic vasculitis leading to increased vascular permeability, edema, hypovolemia and multi-organ failure (Alhassan et al., 2019; Ganta, 2022; Latré De Laté et al., 2025). Rapid intervention is important because the outcome is closely related to the timing of doxycycline administration. In clinical practice, the first signs of the disease are often non-specific and resemble other febrile illnesses resulting in delayed recognition and worse outcomes. The acute phase is particularly problematic to verify in the laboratory (Gottlieb et al., 2018). Serologic assays are commonly performed, though antibody production may not be apparent until 10 to 14 days following exposure and serologic cross-reaction among spotted fever group (SFG) rickettsiae prove difficult to differentiate (Silva et al., 2024). Molecular tests yield direct detection of *R. rickettsii* DNA, but whole-blood sensitivity in the early stages of illness, when organism load is low, can be inconsistent and often quite low. In more advanced or severe disease detection may be enhanced, even though effective antimicrobial therapy can reduce detectable DNA quickly. Specimens based on tissue (e.g., rash biopsy, eschar material) can be expected to result in higher sensitivity, but are not always available to perform diagnostic assessment and qPCR workflows may be challenging where infrastructure is limited (Biggs, 2016; Levin et al., 2016; Stewart and Stewart, 2021). Such limitations underscore the ongoing demand for rapid, simple, and field-deployable molecular diagnostics that can assist clinicians and public health responders in making urgent decisions.

Isothermal amplification platforms are attractive for low-resource environments, as they do not require thermal cycling and can be implemented with minimal instrumentation. Recombinase polymerase amplification (RPA) has been found to be significantly advantageous for point-of-care applications as it operates at low temperature and has rapid kinetics of amplification. When combined with CRISPR/Cas12a, the amplified target is recognized by guide RNA mediated Cas12a complex. After binding to the specific target sequence, Cas12a becomes activated and cleaves a reporter molecule, generating a detectable signal (Hassan et al., 2025). In theory, such a combined RPA–Cas12a structure can improve sequence-specific detection while keeping the workflow rapid and simple (Chen et al., 2018; Hassan et al., 2025; Hueso et al., 2025; Ji et al., 2025; Liu et al., 2025). For RMSF, point-of-care molecular methods are especially relevant because early diagnosis is critical, whereas access to conventional molecular testing may be limited in some clinical or field settings.

Here we developed and tested an isothermal RPA–CRISPR/Cas12a assay for the rapid detection of *R. rickettsii* DNA. We assessed its analytical performance using four *R. rickettsii* strains and related *Rickettsia* species and tested longitudinal samples from dogs experimentally infected with *R. rickettsii* (Sheila Smith and Morgan strains). The study was designed to explore the potential of this platform as a simple and rapid molecular approach for *R. rickettsii* detection and to provide a foundation for future validation in clinically relevant matrices (e.g., removed ticks and lesion-associated materials) and field-amenable sample types.

## 2 Materials and methods

**Figure 1:**
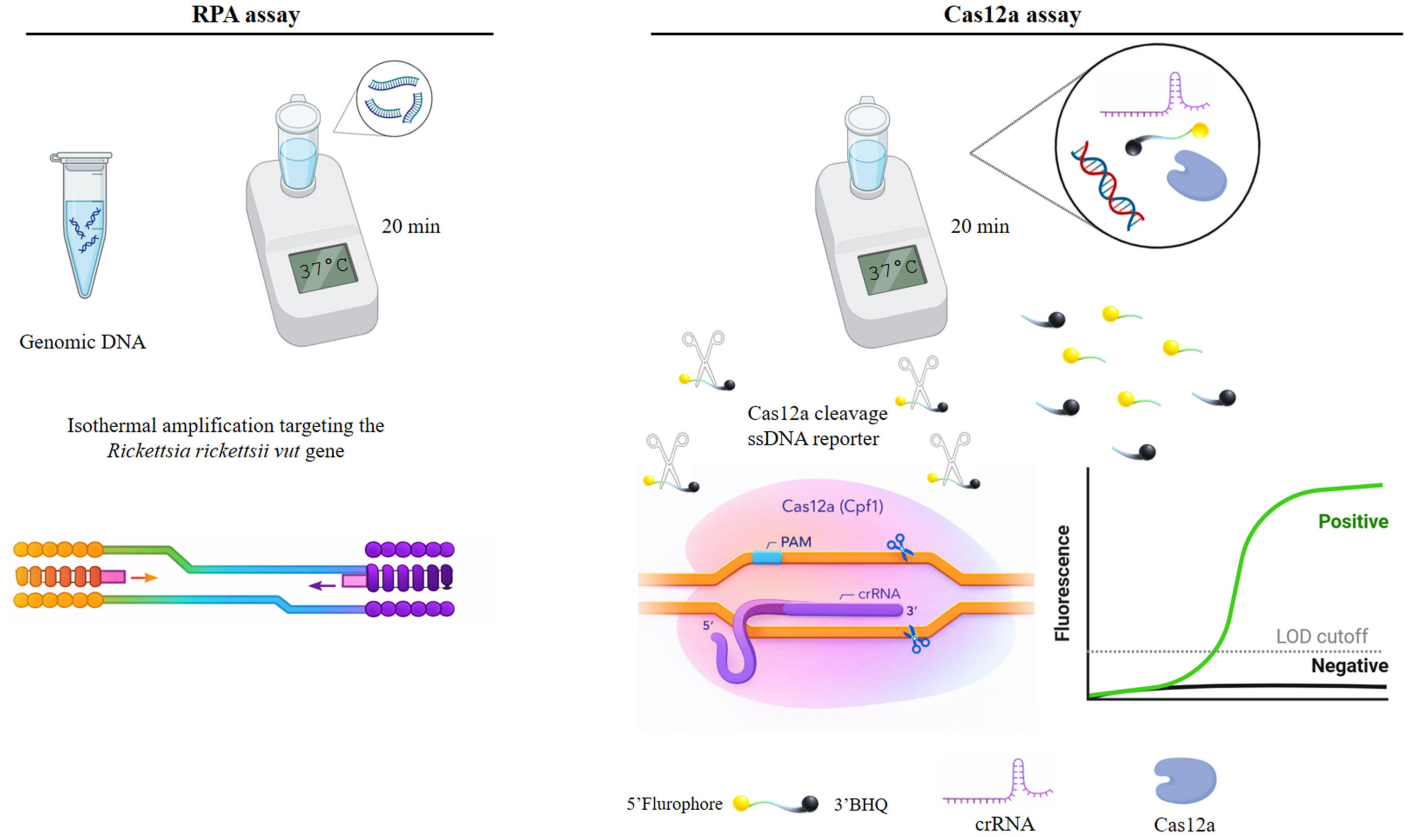
Schematic overview of the RPA–Cas12a assay targeting the *R. rickettsii vut* gene.

The experiment consisted of two consecutive isothermal steps. First, the target molecule was amplified using the RPA method at 37 °C for 20 min. The resulting RPA amplicons were then directly added to the Cas12a detection reaction and incubated for another 20 min at 37 °C in the presence of a fluorophore-quencher single-stranded DNA reporter (Figure 1). Figure 1 was created using BioRender.

### 2.1 Target selection and in silico assay design

*Rickettsia rickettsii vitamin uptake transporter* (*vut*) gene locus (A1G_RS03825; former locus tag A1G_04240; WP_014362924.1) was selected as the molecular target for species-level detection. This gene was selected mainly because it appeared to be suitable for specific detection of *R. rickettsii*. During target screening, we looked for regions that were conserved in available *R. rickettsii* genomes but absent or clearly different in other closely related SFG *Rickettsia* species. Based on these comparisons, the *vut* locus was considered a promising candidate for species-level assay design. Vut family proteins have been identified as transporters involved in salvaging queuosine-related precursors (QPTR), a function consistent with intracellular bacteria that rely on host-derived small molecules (De Crécy-Lagard et al., 2024). To support diagnostic assay specificity, the target region was evaluated *in silico* against nucleotide databases (BLASTn) and by comparative inspection of closely related spotted fever group rickettsiae, focusing on loci reported to be present in *R. rickettsii* but absent or divergent in other *Rickettsia* species. In these *in silico* screens, significant matches were observed for *R. rickettsii* genomes and for the closely related *Rickettsia* sp. CA6269 (also named as *Rickettsia lanei*, sp. nov.) (Probert et al., 2024), whereas no comparable gene homologs were identified among other commonly encountered spotted fever group *Rickettsia* species available in the public databases at the time of this study analysis. Following BLAST based screening, we confirmed the suitability of the selected gene target for nucleic-acid amplification assays.

**Figure 2:**
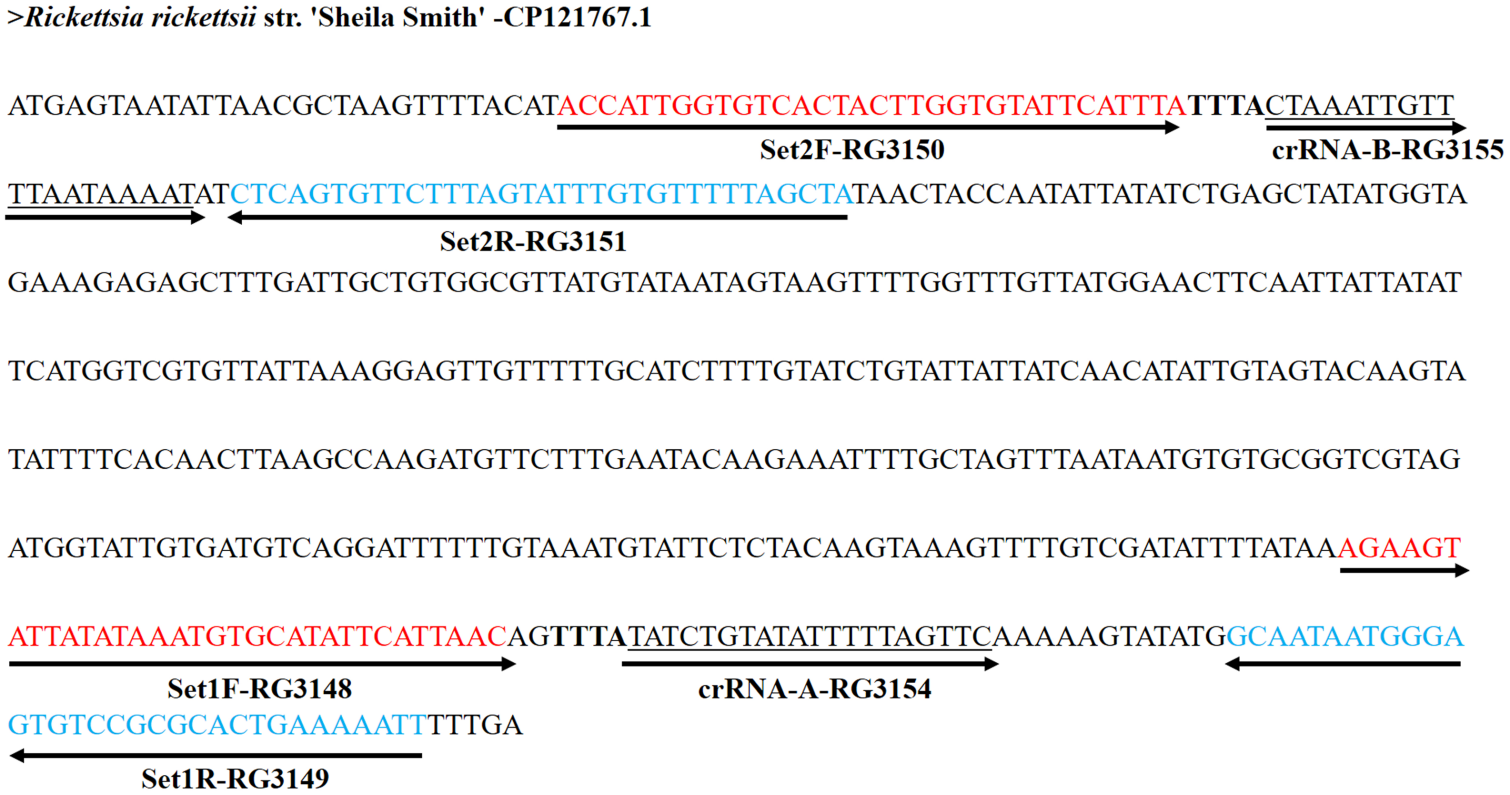
Genomic localization of RPA primers and Cas12a crRNA targets within the *R. rickettsii* Sheila Smith genome (CP121767.1). Forward RPA primers are shown in red, reverse primers in blue, and Cas12a crRNA spacer sequences are underlined. The Cas12a PAM motif is indicated in bold. Set-1 and Set-2 primer–crRNA combinations target distinct regions of the *vut* gene, generating amplicons of 104 bp and 92 bp, respectively.

Two RPA primer sets were designed to amplify short segments within the target locus (104 bp and 92 bp) to support rapid and efficient isothermal amplification (Safenkova et al., 2020) (Table 1). Primer and crRNA design were performed manually based on the selected target sequence, and no dedicated primer/crRNA design software or database was used for this step. For CRISPR-based detection, corresponding crRNAs were designed to provide an additional layer of sequence-specific recognition (Table 2; Figure 2). Guide selection was constrained by the presence of an LbCas12a-compatible PAM (5′-TTTV-3′) within the amplicon and by placement of the spacer to maximize discrimination at the PAM-proximal “seed” region when applicable. Because database searches indicated that the nearest publicly available sequence to this locus belongs to the *Rickettsia* sp. CA6269, we considered *Rickettsia* sp. CA6269 sequence information during guide selection and, designed crRNAs to maximize PAM-proximal discrimination (Supplementary Figure 1). This choice was motivated by published work showing that Cas12a is particularly sensitive to PAM-proximal mismatches (Strohkendl et al., 2018; Kim et al., 2020; Kohabir et al., 2024). We treated this as a conservative design step rather than a demonstrated performance feature, as *Rickettsia* sp. CA6269 reference material was not available for experimental testing.

**Table 1:** Primer design for the *R. rickettsii vut* gene RPA–Cas12a assay.

Primer	Sequence	Length	Amplicon size
Set1F-RG3148	AGAAGTATTATATAAATGTGCATATTCATTAAC	33	104 bp
Set1R-RG3149	AATTTTTCAGTGCGCGGACACTCCCATTATTGC	33	
Set2F-RG3150	ACCATTGGTGTCACTACTTGGTGTATTCATTTA	33	92 bp
Set2R-RG3151	TAGCTAAAAACACAAATACTAAAGAACACTGAG	33	

**Table 2:** crRNA design for the *R. rickettsii vut* gene RPA–Cas12a assay.

crRNA	Sequence	Length
crRNA-A-RG3154	AltR1/rUrArA rUrUrU rCrUrA rCrUrA rArGrU rGrUrA rGrArU rUrArU rCrUrG rUrArU rArUrU rUrUrU rArGrU rUrC/AltR2	41
crRNA-B-RG3155	AltR1/rUrArA rUrUrU rCrUrA rCrUrA rArGrU rGrUrA rGrArU rCrUrA rArArU rUrGrU rUrUrU rArArU rArArA rArU/AltR2	41

### 2.2 Recombinase polymerase amplification (RPA)

Recombinase polymerase amplification (RPA) was carried out using a commercial RPA kit (TwistDx Ltd., Cambridge, UK; cat. no. TABAS03KIT) with target-specific primer set used separately (Set-1: 104 bp; Set-2: 92 bp). RPA reactions were performed in a final volume of 50 μL, containing rehydration buffer, forward and reverse primers, nuclease-free water, and DNA template, with magnesium acetate added to initiate the reaction. Detailed reaction composition is provided in Supplementary Table S1. Recombinase polymerase amplification was performed at 37 °C for 20 min, and to improve amplification homogeneity, tubes were briefly mixed and spun down once during incubation. Following incubation, RPA products were used directly for CRISPR/Cas12a.

### 2.3 Cas12a-based detection and fluorescence readout

Cas12a detection was performed using a commercially available Cas12a enzyme (New England Biolabs, Ipswich, MA, USA; cat. no. M0653S) complexed with a target-specific crRNA designed for each amplicon (Set-1 or Set-2). All primers and crRNAs were synthesized by Integrated DNA Technologies (IDT, Coralville, IA, USA). Cas12a reactions contained Cas12a–crRNA ribonucleoprotein (RNP), reaction buffer supplied by the manufacturer (New England Biolabs, Ipswich, MA, USA; cat. no. M0653S), and Cas12 ssDNA reporter (6-FAM/BHQ1) (SignalChem Diagnostics, Richmond, BC, Canada; cat. no. CS12-57). The final Cas12a reaction volume was 20 μL and included 2 μL of transferred RPA product. For each sample, an aliquot of the RPA product was added to the Cas12a reaction, and mixtures were incubated at 37 °C for 20 min (Supplementary Table S2). Detailed compositions of the RPA and Cas12a reactions, including reagent concentrations and template input, are provided in Supplementary Tables S1, S2.

Fluorescence output from the Cas12a reactions was evaluated using three complementary, FAM-compatible readouts with additional image documentation. For rapid screening, reactions were inspected directly on a UVP transilluminator (Analytik Jena, Jena, Germany) under blue-light illumination; in addition, UV-based endpoint images were captured as needed using a Gel Logic 200 imaging system (Carestream Molecular Imaging, New Haven, CT, USA). For higher-sensitivity documentation, endpoint fluorescence images were also acquired in selected runs using an Amersham™ ImageQuant™ 800 imaging system (Cytiva, Marlborough, MA, USA) with Cy2 channel. Finally, quantitative fluorescence was measured by bottom reading on a microplate reader (Infinite® 200 PRO, Tecan, Männedorf, Switzerland) using black, optical-bottom 96-well plates (Thermo Scientific™ Nunc™; Cat. 165305). Read settings were configured for FAM with excitation at 485 nm and emission at 520 nm (bandwidths 9 nm and 20 nm), 25 flashes per well, and a 20 μs integration time. No-template controls (NTC) were included in every run to monitor background signal and potential contamination.

### 2.4 Preparation of synthetic DNA standards (gBlocks) and serial dilutions

The synthetic double-stranded DNA standards (gBlocks) used for analytical sensitivity experiments were derived from the *R. rickettsii* Sheila Smith *vut* target region, and their sequences are provided in Supplementary Table S3. For analytical sensitivity experiments, synthetic double-stranded DNA fragments covering each target region were prepared (gBlocks; Integrated DNA Technologies, IDT, Coralville, IA, USA) and used as quantitative standards. The lyophilized gBlocks were dissolved in nuclease-free water, and their concentrations were adjusted to 10 ng/μL. The template copy numbers were calculated using Avogadro’s number and the molecular weight provided by the manufacturer: copies/μL = (mass (g/μL) / molecular weight (g/mol)) × 6.022 × 10^23^. Based on these calculations, the resulting stock concentrations were approximately 6.14 × 10^10^ copies/μL for Set 1 and approximately 6.87 × 10^10^ copies/μL for Set 2.

To generate templates across a wide dynamic range, a ten-fold dilution series was prepared in nuclease-free water. Dilutions started at 10^−2^ (2 μL stock + 198 μL water) and were continued by sequential 10-fold steps (10 μL transferred into 90 μL water) through 10^−10^. For limit-of-detection experiments, 1 μL of each dilution was used as input for the RPA reaction. Because the input volume was kept constant, the calculated concentrations (copies/μL) directly reflect the number of copies per reaction. Calculated copy numbers for each dilution are provided in Supplementary Table S4.

### 2.5 Analytical sensitivity and observed limit of detection

The analytical detection limit was evaluated using serial dilutions of synthetic double-stranded DNA (gBlock) standards derived from the *R. rickettsii* Sheila Smith *vut* target region. Three replicate reactions were performed for each dilution (*n* = 3). A reaction was considered positive if the fluorescence exceeded the threshold defined as the mean of the no-template controls plus three standard deviations (NTC mean + 3SD). The positivity threshold was calculated separately for each experimental run using the NTC values generated in that run and was not pooled across experiments. The observed analytical detection limit was reported as the lowest input level that yielded positive results in all replicates (3/3). Because only three replicates were tested per dilution, this observed limit of detection (LOD) should be interpreted as a range-finding estimate rather than an LoD95.

### 2.6 Analytical specificity

This study was designed to assess analytical sensitivity and analytical specificity of the RPA–Cas12a assay and did not include comparison with a clinical gold standard diagnostic method. The analytical specificity was validated by testing genomic DNAs of four *R. rickettsii* strains (Sheila Smith, Morgan, Iowa and Hlp#2) in comparison with a panel of non-target organisms. These organisms comprised spotted fever group rickettsiae (*R. amblyommatis*, *R. montanensis*, *R. philipii* [364D], *R. conorii* and *R. parkeri*), as well as other clinically relevant tick-borne bacteria (*Anaplasma marginale*, *A. phagocytophilum*, *Ehrlichia chaffeensis* and *E. canis*). Genomic DNA of *R. rickettsii* strain Hlp#2, *R. philipii* strain 364D, *R. parkeri* strain Black Gap, *R. conorii* strain Malish 7, *R. amblyommatis* strain WB-8-2, and *R. montanensis* strain ATCC VR-611 were kindly provided by the Division of Vector-Borne Diseases of the Centers for Disease Control and Prevention (CDC), National Center for Zoonotic and Arthropod-Borne Diseases (Atlanta/GA/USA). Each DNA template was assayed with both primer–crRNA Sets 1 and 2 at the same RPA/Cas12a settings. No-template controls (NTC) were included as part of the assay to serve as a negative control. Cross-reactivity was defined as any non-target sample which crossed the positivity cut-off (endpoint fluorescence at 20 min > mean NTC + 3 SD). Each DNA template was tested in triplicate reactions with both primer–crRNA Sets 1 and 2 under the same RPA–Cas12a conditions.

### 2.7 Experimental infection DNA samples (archived extracts) and input into RPA–Cas12a

Archived DNA extracts from experimentally infected Beagle dogs were analyzed in this study which were part of another study (Latré De Laté et al., manuscript in preparation). In the original experiment, dogs were challenged with virulent *R. rickettsii* strains Sheila Smith and Morgan using the infection protocol described previously (Latré De Laté et al., 2025). In the present work, DNA extracts from six dogs were used, including three animals challenged with the Sheila Smith strain (*n* = 3) and three animals challenged with the Morgan strain (*n* = 3), corresponding to days 0, 3, 5, 7, 9, 11, 13, and 15 post-challenge. For this experiment, 2 μL of extracted DNA was added per RPA reaction. All animal procedures were performed in the previously published study (Latré De Laté et al., 2025) under the approved institutional animal care and use protocol described therein; no new animal work was conducted for the present study. Each archived canine blood DNA sample was tested in triplicate reactions at each sampling time point with both primer–crRNA sets.

### 2.8 Statistical analysis

Endpoint fluorescence values obtained in the analytical sensitivity experiment were expressed as mean ± SD from three replicate reactions. Statistical significance relative to the no-template control was assessed using one-way ANOVA followed by Dunnett’s multiple comparisons test. A *p* value < 0.05 was considered statistically significant. The raw endpoint fluorescence values used for figure generation and statistical analysis are provided in the Supplementary Data S1.

## 3 Results

### 3.1 Target selection and assay design

The *R. rickettsii vut* locus was selected as the molecular target for experimental development based on *in silico* screening supporting its conservation within *R. rickettsii* and its differentiation from closely related non-target SFG Rickettsia species. Comparative sequence analysis identified the closest publicly available sequence to this target region as Rickettsia sp. CA6269. Two sets of RPA primers generating 104 bp and 92 bp amplicons, along with their corresponding crRNAs, were selected for RPA–Cas12a assay (Tables 1, 2; Figure 2).

### 3.2 Assay validation using *Rickettsia rickettsii* strains

**Figure 3:**
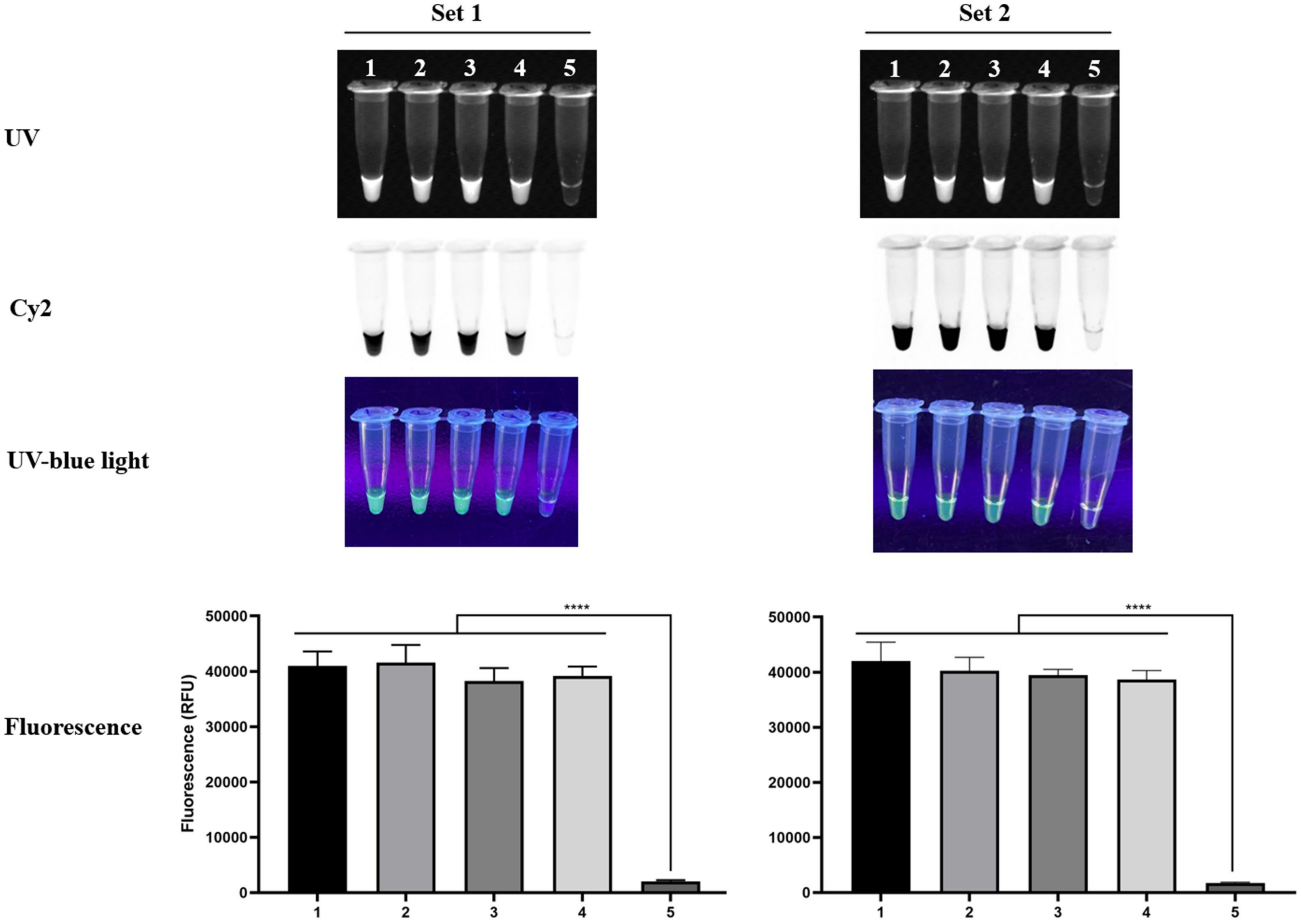
Validation of the RPA–Cas12a assay using four *R. rickettsii* strains (Set 1 and Set 2). RPA was performed at 37 °C for 20 min, followed by Cas12a detection at 37 °C for 20 min. Representative endpoint reaction images are shown under UV, Cy2, and UV-blue light. Quantitative fluorescence values are shown as mean ± SD from triplicate reactions. Sample order (1–5): Sheila Smith, Morgan, Iowa, Hlp#2, and no-template control (NTC).

First, we confirmed that the RPA–Cas12a workflow reliably detected four different *R. rickettsii* strains using both primer–crRNA designs (Set 1 and Set 2). Under standard two-step isothermal conditions (20 min of RPA at 37 °C followed by 20 min of Cas12a detection at 37 °C), clear positive signals were observed for the *R. rickettsii* strains: Sheila Smith, Morgan, Iowa, and Hlp#2 strains, while the no-template control remained negative in all readings (Figure 3). Consistent results were obtained with both direct tube visualization (UV/blue light and Cy2/FAM compatible imaging) and microplate-based fluorescence quantification, supporting that the test result is easily interpretable and remains consistent with quantitative documentation (Figure 3).

### 3.3 Analytical sensitivity and observed limit of detection using serial dilutions

**Figure 4:**
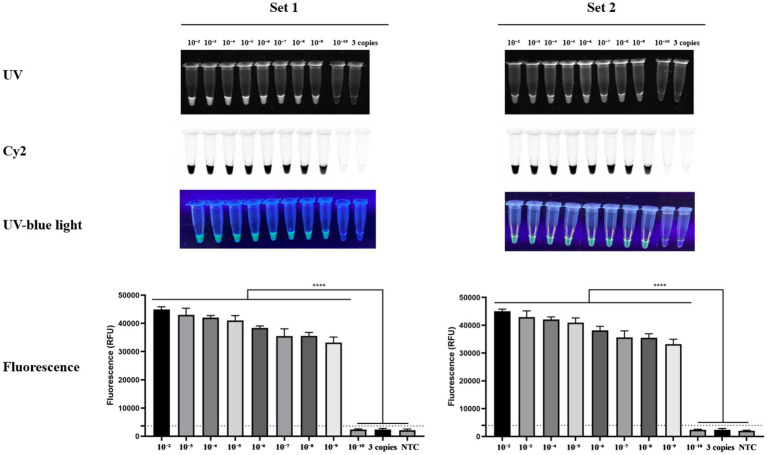
Analytical sensitivity and observed limit of detection of the RPA–Cas12a assay targeting the *R. rickettsii vut* gene. Serial 10-fold dilutions of *R. rickettsii* gBlock DNA (10^−2^–10^−10^) and a low-copy control (3 copies) were tested in two independent experimental sets (Set 1 and Set 2). RPA was performed at 37 °C for 20 min followed by Cas12a detection at 37 °C for 20 min. Reaction outputs are shown under UV, Cy2, and UV-blue light, with corresponding endpoint RFU measurements (mean ± SD; *n* = 3). The positivity threshold was defined as mean NTC + 3 SD; the observed LOD corresponds to the lowest input level positive in all replicates (3/3). Statistical significance was determined by one-way ANOVA with Dunnett’s multiple comparisons test (****, *p* < 0.0001).

We compared analytical sensitivity across a wide working range using 10-fold serial dilutions of synthetic double-stranded DNA (gBlock) standards derived from the *R. rickettsii* Sheila Smith *vut* target region. Dilutions were evaluated in two separate experimental blocks corresponding to the two primer–crRNA pairs (Set 1 and Set 2), with three replicate reactions per dilution (*n* = 3). As expected, endpoint fluorescence decreased with decreasing template input; however, signals remained well-defined relative to the no-template controls across much of the dilution series (Figure 4). A reaction was considered positive if the fluorescence exceeded the positivity threshold defined as the mean of the no-template controls plus three standard deviations (NTC mean + 3SD). Based on the copy number determination of the dilution series (Supplementary Table S4), the lowest input level that remained positive in all replicates (3/3) was 10^−9^, corresponding to approximately 6.1 × 10^1^ copies per reaction for Set 1 and 6.9 × 10^1^ copies per reaction for Set 2. Collectively, these results place the assay’s observed analytical detection limit at ~60–70 copies per reaction and indicate that the integrated RPA–Cas12a workflow provides a rapid readout with a high signal-to-background ratio, supporting further validation in biological matrices.

### 3.4 Analytical specificity against non-target rickettsiae and tick-borne bacteria

**Figure 5:**
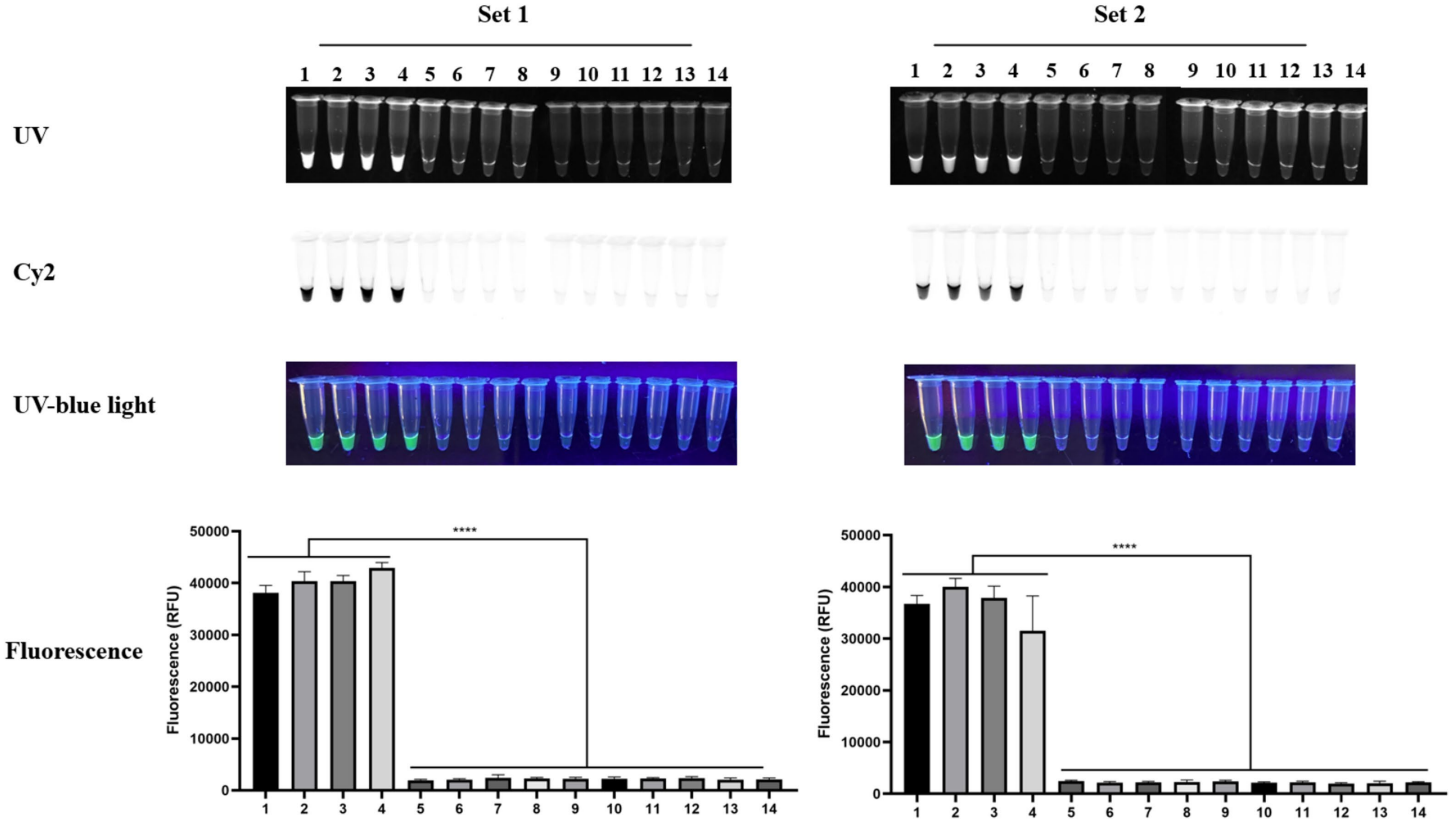
Specificity of the RPA–Cas12a assay targeting *R. rickettsii*. Lanes: 1, *R. rickettsii* (Sheila Smith); 2, *R. rickettsii* (Morgan); 3, *R. rickettsii* (Iowa); 4, *R. rickettsii* (Hlp#2); 5, *R. amblyommatis*; 6, *R. montanensis*; 7, *R. philipii* (364D); 8, *R. conorii*; 9, *R. parkeri*; 10, *Anaplasma marginale*; 11, *A. phagocytophilum*; 12, *Ehrlichia chaffeensis*; 13, *E. canis*; 14, no-template control (NTC). Endpoint fluorescence values are shown as mean ± SD from triplicate reactions.

Analytical specificity was evaluated using a panel of closely related spotted fever group rickettsiae and other clinically relevant tick-borne bacterial DNAs as templates. While strong positive signals were observed for the four *R. rickettsii* strains (lanes 1–4), non-target rickettsiae (*R. amblyommatis*, *R. montanensis*, *R. philipii* [364D], *R. conorii*, and *R. parkeri*) and non-rickettsial tick-borne bacterial DNAs (*A. marginale*, *A. phagocytophilum*, *E. chaffeensis*, and *E. canis*) remained at background levels and did not exceed the positivity threshold for either Set 1 or Set 2. The no-template control was consistently negative. Together, these findings support the specificity of the *vut* gene targeted assay within the tested panel and demonstrate no detectable cross-reactivity under the reaction conditions used (Figure 5).

### 3.5 Detection of *Rickettsia rickettsii* DNA in archived longitudinal canine blood extracts

**Figure 6:**
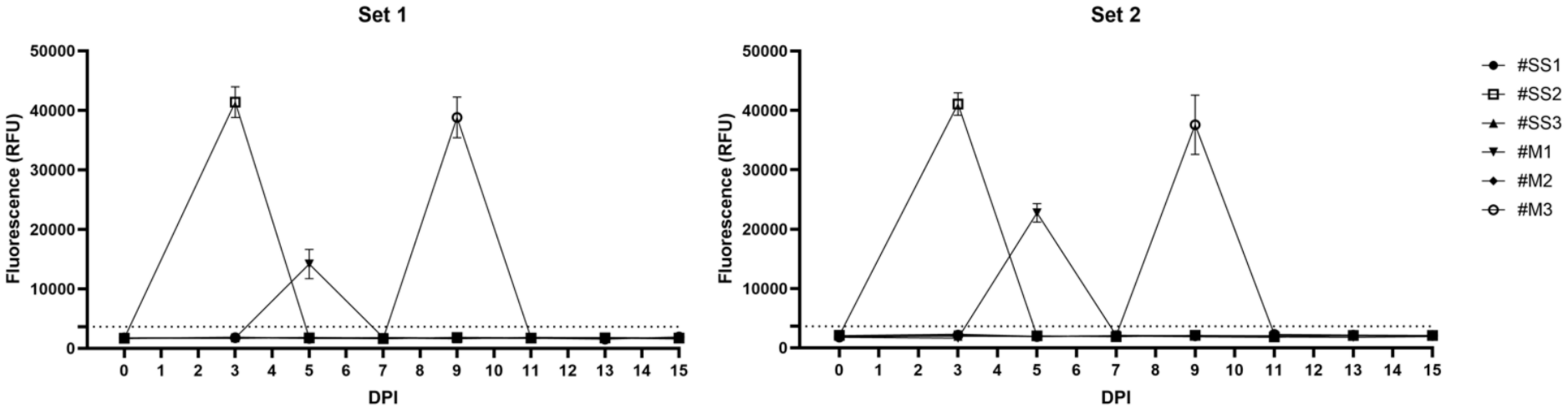
Detection of *R. rickettsii* DNA in archived longitudinal canine blood DNA extracts using the RPA–Cas12a assay (Set 1 and Set 2). Archived DNA extracts from experimentally infected beagle dogs challenged with Sheila Smith strain (#SS1–#SS3; *n* = 3) or the Morgan strain (#M1–#M3; *n* = 3) were tested at the indicated days post-infection (DPI) using two independent primer–crRNA designs. Each time point was tested in triplicate reactions, and the plotted values represent mean endpoint fluorescence (RFU) ± SD from the Cas12a step. The dashed line represents the same fluorescence decision threshold used for the positivity calls (NTC mean + 3 SD).

We next applied the assay to archived DNA samples obtained from experimentally infected Beagle dogs sampled longitudinally after infection with virulent *R. rickettsii* Sheila Smith and Morgan strains. The canine blood samples were collected as part of another study and DNAs recovered from the samples were utilized in the current study for defining the value of the diagnostic assay for *in vivo* sample application. Over the 0 to 15-day sampling period from the infected dogs, detection occurred intermittently and not continuously, at selected time points. In the Sheila Smith group, one dog yielded a positive signal on day 3 post-infection, while in the Morgan group, two dogs tested positive, one on day 5 and the other on day 9. Importantly, two independent primer and crRNA designs produced the same consistent results for these DNA extracts; the same animals scored positive on the same sampling days in both Set 1 and Set 2, and no timing differences were observed between the sets (Figure 6). These findings provide fundamental evidence that the rapid RPA–Cas12a method can detect *R. rickettsii* in DNA obtained from canine whole blood samples but also show that positive results were limited to specific sampling days rather than being consistently present throughout the 0–15 day period.

## 4 Discussion

CRISPR/Cas12a-based assays have been increasingly used for rapid and sequence-specific pathogen detection, especially when combined with isothermal amplification methods such as RPA (Chen et al., 2018; Hassan et al., 2025; Hueso et al., 2025; Ji et al., 2025; Liu et al., 2025). These systems use a guide RNA to recognize the target sequence, and once the target is recognized, Cas12a cleaves a reporter molecule to generate a detectable signal. This study establishes a rapid and fully isothermal RPA–CRISPR/Cas12a workflow for detecting *R. rickettsii*, yielding results in approximately 40 min at 37 °C, and demonstrating consistent performance with two independent primer–crRNA designs. While the same genomic region has previously been used for RPA–lateral flow detection with BLAST-supported specificity screening (Qi et al., 2018), our work expands upon this foundation by utilizing the guide-directed Cas12a readout and included two sequence-specific recognition steps, which substantially increases the specificity of the pathogen detection and moreover, improves the interpretability of results without increasing equipment requirements. We evaluated specificity using a panel of four different *R. rickettsii* strains and several non-target organisms relevant to RMSF-like symptoms and tick-borne co-infections. The assay generated a signal only with *R. rickettsii* templates and showed no detectable cross-reactivity with the non-target organisms tested. The fluorescent format supports both rapid visual readouts under blue/UV light and standardized documentation using a plate reader.

Comparative *in silico* screening showed that close matches exhibiting high similarity to the *R. rickettsii vut* gene were largely limited to the lineage associated with *Rickettsia* sp. CA6269 (*Rickettsia lanei*). Therefore, this sequence was considered during crRNA design. Accordingly, *Rickettsia* sp. CA6269 was considered the primary close neighbor, and where possible, crRNAs were designed to include a mismatch near the PAM as a conservative design measure based on published Cas12a seed/PAM mismatch sensitivity (Strohkendl et al., 2018; Kim et al., 2020; Kohabir et al., 2024). However this feature was not experimentally validated due to the unavailability of *Rickettsia* sp. CA6269 reference material.

Throughout the 10-fold dilution series in our study, positive reactions remained clearly distinguishable from template-free controls across a wide dynamic range, and the practical limit of detection was in the tens of copies per reaction (~60–70 copies per reaction under our conditions). This level of analytical sensitivity is generally consistent with those reported for other RPA/ERA–Cas12a workflows applied to tick-borne pathogens and demonstrates how strongly the apparent LOD depends on the template format, reporting unit, and sample matrix (Paenkaew et al., 2023, 2024, 2026; Feng et al., 2025). For example, an RPA–Cas12a assay for *A. marginale* reported detection down to 4 copies/μL, offering both fluorescence and lateral flow readouts, highlighting its field applicability (Sutipatanasomboon et al., 2024). In the diagnosis of *Hepatozoon canis* in dogs, an RPA–Cas12a assay reported about 10 copy detection using plasmid standards per reaction, but the practical limit increased when evaluated under conditions spiked with target blood (~100 copies per reaction), underscoring the expected inhibitory/partitioning effects of blood matrices even when amplification is isothermal (Paenkaew et al., 2024). Similarly, an RPA–Cas12a approach for *E. canis* and *Anaplasma platys* defined the sensitivity at approximately 100 copies (Paenkaew et al., 2023). An ERA-Cas12a system for *Theileria annulata*, an important blood protozoan of cattle, reported a sensitivity of about 10 copies/μL with an approximately 40-min workflow, again highlighting that the numerical LOD depends on whether the results are expressed per microliter, per reaction, or in matrix-mixed conditions (Feng et al., 2025). At the same time, established qPCR and ddPCR-based methods can provide stronger quantitative performance for sensitive molecular detection. In this sense, the current test should not be seen as a method to replace standard qPCR workflows at this stage. Rather, its main practical advantage lies in its simplicity: it operates entirely under isothermal conditions, provides results in approximately 40 min, and requires less complex equipment; this can make it useful as a rapid complementary platform where conventional PCR infrastructure is limited or where rapid results are a priority. Overall, the observed analytical detection limit in the 60–70 copies range is within the expected range for blood-based isothermal CRISPR diagnostics, and the inclusion of two independent primer-crRNA designs provides practical robustness in case PAM constraints or primer behavior make any single amplicon window less stable across different sample types.

Using archived blood DNA samples, we detected *R. rickettsii* intermittently, not continuously. Only 3 out of 6 dogs yielded a positive signal, and positivity was limited to specific sampling points (days 3–5 and day 9 post-infection). These dogs developed a clinical picture consistent with acute RMSF after experimental infection, including fever, lethargy, anorexia, and hematological changes such as thrombocytopenia (clinical observations recorded during the experimental study). However, molecular detection from whole blood did not consistently track these clinical features on a daily basis. This pattern is biologically plausible given the endothelial tropism of *R. rickettsii* and the resulting low-level, transient, and spatially heterogeneous rickettsemia, which can make whole blood PCR positivity sporadic even in controlled infections (Piranda et al., 2008, 2011; Alhassan et al., 2019; Latré De Laté et al., 2025). Previous experimental studies have similarly reported that blood-based DNA detection tends to occur within a relatively narrow window after inoculation or tick infestation, and its duration can subsequently vary; this supports the practical interpretation that a negative blood PCR at a single time point does not rule out infection (Piranda et al., 2008). In an experimentally infected *Dermacentor variabilis* tick-challenge model, venous blood PCR was intermittently positive and not consistently aligned with fever, highlighting the value of time-structured sampling (Levin et al., 2014). Comparable results are also obtained from canine vaccination/infection and low-dose longitudinal studies, where blood qPCR/PCR positivity may be transient, while tissue testing and/or seroconversion provide broader evidence of infection burden (Alhassan et al., 2019). Specifically, even under controlled infection conditions, it has been reported that in most dogs, blood-based nested PCR was positive on only a single sampling day, with surrounding time points being negative; this highlights how narrow and inconsistent the detection window in EDTA blood can be (Alhassan et al., 2019; Latré De Laté et al., 2025). Accordingly, the current blood-based findings should be interpreted as preliminary biological proof-of-principle data rather than as evidence of clinical sensitivity in whole-blood testing.

These observations have practical implications for both diagnosis and surveillance. In dogs, relying on whole blood as the primary molecular matrix may underestimate infection or exposure, as a negative result at a single time point may reflect transient, heterogeneous rickettsiemia rather than true absence of infection. In this context, a rapid isothermal workflow should be viewed as a rapid molecular platform with potential for further development rather than as a fully validated field-screening tool. Although we did not evaluate ticks, lesion-associated materials, conjunctival swabs, or other field-collected samples in the present study, these remain reasonable directions for future validation. However, performance in those sample types will need to be established through additional validation. Future work should therefore focus on testing the assay in a broader range of clinically relevant samples together with simple, field-friendly extraction and readout formats.

## 5 Conclusion

We developed a rapid and fully isothermal RPA–CRISPR/Cas12a assay targeting the *R. rickettsii vut* gene locus region. This assay provides results in approximately 40 min at 37 °C and demonstrates consistent performance with two independent primer-crRNA designs. Testing using quantified synthetic standards and a panel of organisms demonstrated sensitive detection, and no detectable cross-reactivity within the test panel was observed under the tested conditions. Intermittent detections from longitudinally collected whole blood samples in experimentally infected dogs confirm a known limitation of using blood as the primary molecular matrix for RMSF. At this stage, the assay is better viewed as a rapid molecular platform for complementary testing rather than as a fully defined screening test. Overall, these findings support the feasibility of the assay as a rapid isothermal molecular platform, while indicating that broader validation in additional clinically relevant sample types (e.g., lesion-associated materials) and removed ticks is still needed.

## Data Availability

The original contributions presented in the study are included in the article/Supplementary material, further inquiries can be directed to the corresponding authors.

## Supplementary material

SUPPLEMENTARY DATA S1 Raw RFU values used for analytical sensitivity, specificity, and archived canine blood sample analyses (file: Supplementary_Data_Raw_RFU_Values.xlsx).

SUPPLEMENTARY TABLE S1 Tables S1 to S4.

SUPPLEMENTARY DATA SHEET S1 Raw data file.

SUPPLEMENTARY DATA IMAGE S1 Figure S1.

## References

[ref1] Alhassan A. Liu H. McGill J. Cerezo A. Jakkula L. U. M. R. Nair A. D. S. . (2019). *Rickettsia rickettsii* whole-cell antigens offer protection against Rocky Mountain spotted fever in the canine host. Infect. Immun. 87, e00628–e00618. doi: 10.1128/IAI.00628-18, 30396898 PMC6346123

[ref2] Álvarez-Hernández G. Paddock C. D. Walker D. H. Valenzuela J. G. Calleja-López J. T. Rivera-Rosas C. N. . (2024). Rocky Mountain spotted fever is a neglected tropical disease in Latin America. PLoS Negl. Trop. Dis. 18:e0012276. doi: 10.1371/journal.pntd.0012276 38990838 PMC11238974

[ref3] Álvarez-Hernández G. Roldán J. F. G. Milan N. S. H. Lash R. R. Behravesh C. B. Paddock C. D. (2017). Rocky Mountain spotted fever in Mexico: past, present, and future. Lancet Infect. Dis. 17, e189–e196. doi: 10.1016/S1473-3099(17)30173-1, 28365226

[ref4] Biggs H. M. (2016). Diagnosis and management of tickborne rickettsial diseases: Rocky Mountain spotted fever and other spotted fever group rickettsioses, ehrlichioses, and anaplasmosis—United States. MMWR Recomm. Rep. 65, 1–44. doi: 10.15585/mmwr.rr6502a1, 27172113

[ref5] Brophy M. K. Drexler N. A. Stone N. E. Busch J. D. Ballard R. Bourgeois R. M. . (2025). Ecologic risk factors for infestation of *Rhipicephalus sanguineus* sl in a Rocky Mountain spotted fever-endemic area of eastern Arizona. Am. J. Trop. Med. Hyg. 113:156. doi: 10.4269/ajtmh.24-0485, 40328244 PMC12225560

[ref6] Chen J. S. Ma E. Harrington L. B. Da Costa M. Tian X. Palefsky J. M. . (2018). CRISPR-Cas12a target binding unleashes indiscriminate single-stranded DNase activity. Science 360, 436–439. doi: 10.1126/science.aar6245, 29449511 PMC6628903

[ref7] De Crécy-Lagard V. Hutinet G. Cediel-Becerra J. D. D. Yuan Y. Zallot R. Chevrette M. G. . (2024). Biosynthesis and function of 7-deazaguanine derivatives in bacteria and phages. Microbiol. Mol. Biol. Rev. 88:e0019923-23. doi: 10.1128/mmbr.00199-23, 38421302 PMC10966956

[ref8] Feng X. Li Y. Li S. Zafar I. Rizk M. A. Fu X. . (2025). Rapid and sensitive detection of bovine Theileria annulata parasite based on ERA-CRISPR/Cas12a technology. Front. Microbiol. 16:1647929. doi: 10.3389/fmicb.2025.1647929, 40831638 PMC12358460

[ref9] Ganta R. R. (2022). “Rickettsiaceae and Coxiellaceae: Rickettsia and Coxiella,” in Veterinary Microbiology, eds. McVey D. S. Kennedy M. Chengappa M. M. Wilkes R. (New York: Wiley), 377–380.

[ref10] Gottlieb M. Long B. Koyfman A. (2018). The evaluation and management of Rocky Mountain spotted fever in the emergency department: a review of the literature. J. Emerg. Med. 55, 42–50. doi: 10.1016/j.jemermed.2018.02.043, 29685474

[ref11] Hassan Y. M. Mohamed A. S. Hassan Y. M. El-Sayed W. M. (2025). Recent developments and future directions in point-of-care next-generation CRISPR-based rapid diagnosis. Clin. Exp. Med. 25:33. doi: 10.1007/s10238-024-01540-8, 39789283 PMC11717804

[ref12] Hueso L. Martorell S. Sena-Torralba A. Ferrando M. Ferri M. Maquieira A. . (2025). Recombinase polymerase amplification technology for point-of-care diagnosis of neglected tropical diseases. Int. J. Infect. Dis. 153:107831. doi: 10.1016/j.ijid.2025.107831, 39900222

[ref13] Ji T. Fang X. Gao Y. Yu K. Gao X. (2025). Research progress on the application of RPA-CRISPR/Cas12a in the rapid visual detection of pathogenic microorganisms. Front. Cell. Infect. Microbiol. 15:1640938. doi: 10.3389/fcimb.2025.1640938, 40809520 PMC12343600

[ref14] Kidd L. Breitschwerdt E. B. (2013). “Rocky Mountain spotted fever” in Canine and Feline Infectious Diseases. Ed. Sykes, J. E. (St Louis, MO: Elsevier Saunders), p. 300–310.

[ref15] Kim H. Lee W. Oh Y. Kang S.-H. Hur J. K. Lee H. . (2020). Enhancement of target specificity of CRISPR–Cas12a by using a chimeric DNA–RNA guide. Nucleic Acids Res. 48, 8601–8616. doi: 10.1093/nar/gkaa605, 32687187 PMC7470973

[ref16] Kohabir K. A. Linthorst J. Nooi L. O. Brouwer R. Wolthuis R. M. Sistermans E. A. (2024). Synthetic mismatches enable specific CRISPR-Cas12a-based detection of genome-wide SNVs tracked by ARTEMIS. Cell Rep. Methods 16:100912. doi: 10.1016/j.crmeth.2024.100912, 39644903 PMC11704620

[ref17] Labruna M. B. Kamakura O. Moraes-Filho J. Horta M. C. Pacheco R. C. (2009). Rocky mountain spotted fever in dogs, Brazil. Emerg. Infect. Dis. 15, 458–460. doi: 10.3201/eid1503.081227, 19239764 PMC2681129

[ref18] Latré De Laté P. Stoll I. M. Ferm J. Madesh S. Ferm D. Chauhan D. . (2025). *Rickettsia rickettsii* inactivated whole cell antigen vaccine protects against Rocky Mountain spotted fever independent of the adjuvant used. Infect. Immun. 93:e0041225. doi: 10.1128/iai.00412-25, 41159782 PMC12707144

[ref19] Laukaitis H. J. Macaluso K. R. (2021). Unpacking the intricacies of *Rickettsia*–vector interactions. Trends Parasitol. 37, 734–746. doi: 10.1016/j.pt.2021.05.008, 34162522 PMC8344978

[ref20] Levin M. L. Killmaster L. F. Zemtsova G. E. Ritter J. M. Langham G. (2014). Clinical presentation, convalescence, and relapse of Rocky Mountain spotted fever in dogs experimentally infected via tick bite. PLoS One 9:e115105. doi: 10.1371/journal.pone.0115105, 25542001 PMC4277292

[ref21] Levin M. L. Snellgrove A. N. Zemtsova G. E. (2016). Comparative value of blood and skin samples for diagnosis of spotted fever group rickettsial infection in model animals. Ticks Tick-Borne Dis. 7, 1029–1034. doi: 10.1016/j.ttbdis.2016.05.011, 27282078 PMC5661872

[ref22] Liu M. Hou Y. Cheng Y. Li Z. Zeng J. Li L. . (2025). Recent advances in isothermal amplification techniques coupled with clustered regularly interspaced short palindromic repeat/Cas systems. Interdiscip. Med. 3:e20250052. doi: 10.1002/INMD.20250052

[ref23] Nicholson W. L. Allen K. E. McQuiston J. H. Breitschwerdt E. B. Little S. E. (2010). The increasing recognition of rickettsial pathogens in dogs and people. Trends Parasitol. 26, 205–212. doi: 10.1016/j.pt.2010.01.007, 20207197

[ref24] Paenkaew S. Euppayo T. Tungtrakanpoung R. Teapunvong W. Nganvongpanit K. Buddhachat K. (2026). Rapid and specific detection of *Babesia vogeli* using RPA/CRISPR-Cas12a: a feasible field-friendly diagnostic for canine babesiosis. Vet. Parasitol. 342:110660. doi: 10.1016/j.vetpar.2025.110660, 41338106

[ref25] Paenkaew S. Jaito N. Pradit W. Chomdej S. Nganvongpanit K. Siengdee P. . (2023). RPA/CRISPR-cas12a as a specific, sensitive and rapid method for diagnosing *Ehrlichia canis* and *Anaplasma platys* in dogs in Thailand. Vet. Res. Commun. 47, 1601–1613. doi: 10.1007/s11259-023-10114-0, 36997812 PMC10062689

[ref26] Paenkaew S. Poommouang A. Pradit W. Chomdej S. Nganvongpanit K. Siengdee P. . (2024). Feasibility of implementing RPA coupled with CRISPR-Cas12a (RPA-Cas12a) for *Hepatozoon canis* detection in dogs. Vet. Parasitol. 331:110298. doi: 10.1016/j.vetpar.2024.110298, 39217761

[ref27] Piranda E. M. Faccini J. L. H. Pinter A. Pacheco R. C. Cançado P. H. D. Labruna M. B. (2011). Experimental infection of *Rhipicephalus sanguineus* ticks with the bacterium *Rickettsia rickettsii*, using experimentally infected dogs. Vector-Borne Zoonotic Dis. 11, 29–36. doi: 10.1089/vbz.2009.0250, 20569011

[ref28] Piranda E. M. Faccini J. L. H. Pinter A. Saito T. B. Pacheco R. C. Hagiwara M. K. . (2008). Experimental infection of dogs with a Brazilian strain of *Rickettsia rickettsii*: clinical and laboratory findings. Mem. Inst. Oswaldo Cruz 103, 696–701. doi: 10.1590/S0074-02762008000700012, 19057821

[ref29] Probert W. S. Haw M. P. Nichol A. C. Glaser C. A. Park S. Y. Campbell L. E. . (2024). Newly recognized spotted fever group *Rickettsia* as cause of severe Rocky Mountain spotted fever–like illness, northern California, USA. Emerg. Infect. Dis. 30:1344. doi: 10.3201/eid3007.231771, 38816345 PMC11210658

[ref30] Qi Y. Shao Y. Rao J. Shen W. Yin Q. Li X. . (2018). Development of a rapid and visual detection method for *Rickettsia rickettsii* combining recombinase polymerase assay with lateral flow test. PLoS One 13:e0207811. doi: 10.1371/journal.pone.0207811, 30475889 PMC6257923

[ref31] Rubino F. Foley P. Foley J. (2025). Control of *Rhipicephalus sanguineus* ticks and Rocky Mountain spotted fever informed by an in silico tool. Infect. Dis. Model. 10, 1179–1189. doi: 10.1016/j.idm.2025.06.005, 40678792 PMC12270066

[ref32] Safenkova I. V. Ivanov A. V. Slutskaya E. S. Samokhvalov A. V. Zherdev A. V. Dzantiev B. B. (2020). Key significance of DNA-target size in lateral flow assay coupled with recombinase polymerase amplification. Anal. Chim. Acta 1102, 109–118. doi: 10.1016/j.aca.2019.12.048, 32043989

[ref33] Sánchez Pérez M. Feria Arroyo T. P. Venegas Barrera C. S. Sosa-Gutiérrez C. Torres J. Brown K. A. . (2023). Predicting the impact of climate change on the distribution of *Rhipicephalus sanguineus* in the Americas. Sustainability 15, 1–12. doi: 10.3390/su15054557, 41704547 PMC7618726

[ref34] Silva K. A. Prado V. B. do Silva R. R. MVP Rocha de Oliveira R. A. R. Falcão T. de J. R. (2024). A mini-review of diagnostic methods for the antigen and antibody detection of Rocky Mountain and Brazilian spotted fever. Biomedicine 12:1501 doi: 10.3390/biomedicines12071501. PMC11274458 39062074

[ref35] Stewart A. G. Stewart A. G. (2021). An update on the laboratory diagnosis of *Rickettsia* spp. infection. Pathogens 10:1319. doi: 10.3390/pathogens10101319, 34684267 PMC8541673

[ref36] Strohkendl I. Saifuddin F. A. Rybarski J. R. Finkelstein I. J. Russell R. (2018). Kinetic basis for DNA target specificity of CRISPR-Cas12a. Mol. Cell 71, 816–824.e3. doi: 10.1016/j.molcel.2018.06.043, 30078724 PMC6679935

[ref37] Sutipatanasomboon A. Wongsantichon J. Sakdee S. Naksith P. Watthanadirek A. Anuracpreeda P. . (2024). RPA-CRISPR/Cas12a assay for the diagnosis of bovine *Anaplasma marginale* infection. Sci. Rep. 14:7820. doi: 10.1038/s41598-024-58169-6, 38570576 PMC10991388

[ref38] Wolbach S. B. (1919). Studies on Rocky Mountain spotted fever. J. Med. Res. 41:1. 19972499 PMC2104421

