# Supplementary material for: A rapid isothermal RPA–CRISPR/Cas12a assay for detection of Rickettsia rickettsii
Source: Front Microbiol. 2026 Apr 16;17:1823193. doi: 10.3389/fmicb.2026.1823193 (PMC13128586; doi:10.3389/fmicb.2026.1823193)
Supplement: SUPPLEMENTARY DATA S1 — Raw RFU values used for analytical sensitivity, specificity, and archived canine blood sample analyses (file: Supplementary_Data_Raw_RFU_Values.xlsx). [file Table_1.DOCX]

Supplementary Material

**Supplementary Table S1:** RPA reaction composition (50 µL).

| **Component** | **Volume** |
| --- | --- |
| Rehydration Buffer | 29.5 µL |
| Forward primer (10 µM) | 2.4 µL |
| Reverse primer (10 µM) | 2.4 µL |
| Nuclease-free water (NFW) | 13.2 µL |
| DNA template (gblock 1 µL**)** | 2.5 µL |
| **Total** | **50** µL |
| Magnesium acetate (MgOAc) | 2 µL (Added to cap; initiates reaction) |

**Supplementary Table S2:** Cas12a reaction composition (20 µL).

| **Component** | **Volume** |
| --- | --- |
| 10× NEBuffer | 2 µL |
| EnGen LbCas12a (Cpf1) (1 µM) | 1 µL |
| crRNA (10 µM) | 0.2 µL |
| Nuclease-free water (NFW) | 14.4 µL |
| Fluorescent reporter (FAM–BHQ1) | 0.4 µL |
| RPA product | 2 µL |
| **Total** | **20** µL |

**Supplementary Table S3:** Sequences of the synthetic DNA standards (gBlocks) used for analytical sensitivity testing.

| **Synthetic DNA standard** | **Reference sequence** | **Sequence (5′–3′)** |
| --- | --- | --- |
| Set 1 gBlock (*R. rickettsii*, 158 bp) | Derived from the *R. rickettsii* Sheila Smith *vut* target region (CP121767.1) | GATCGTACGATGACCTGATCGTAGCAGAAGTATTATATAAATGTGCATATTCATTAACAGTTTATATCTGTATATTTTTAGTTCAAAAAGTATATGGCAATAATGGGAGTGTCCGCGCACTGAAAAATTTTGACTAGTCGATGACGTCATCGATGCAT |
| Set 2 gBlock (*R. rickettsii*, 142 bp) | Derived from the *R. rickettsii* Sheila Smith *vut* target region (CP121767.1) | GATCGTACGATGACCTGATCGTAGCACCATTGGTGTCACTACTTGGTGTATTCATTTATTTACTAAATTGTTTTAATAAAATATCTCAGTGTTCTTTAGTATTTGTGTTTTTAGCTACTAGTCGATGACGTCATCGATGCAT |

**Supplementary Table S4:** Calculated template copy numbers used for the limit-of-detection (LOD) analysis (gBlock serial dilutions).

| **Dilution** | **Set 1 (stock = 6.14×10¹⁰ copies/µL)** | **Set 2 (stock = 6.87×10¹⁰ copies/µL)** |
| --- | --- | --- |
| 10⁻² | 6.14×10⁸ | 6.87×10⁸ |
| 10⁻³ | 6.14×10⁷ | 6.87×10⁷ |
| 10⁻⁴ | 6.14×10⁶ | 6.87×10⁶ |
| 10⁻⁵ | 6.14×10⁵ | 6.87×10⁵ |
| 10⁻⁶ | 6.14×10⁴ | 6.87×10⁴ |
| 10⁻⁷ | 6.14×10³ | 6.87×10³ |
| 10⁻⁸ | 6.14×10² | 6.87×10² |
| 10⁻⁹ | 6.14×10¹ | 6.87×10¹ |
| 10⁻¹⁰ | 6.14×10⁰ (**≈ 6.14**) | 6.87×10⁰ (**≈ 6.87**) |
