# Supplementary material for: A rapid isothermal RPA–CRISPR/Cas12a assay for detection of Rickettsia rickettsii
Source: Front Microbiol. 2026 Apr 16;17:1823193. doi: 10.3389/fmicb.2026.1823193 (PMC13128586; doi:10.3389/fmicb.2026.1823193)
Supplement: SUPPLEMENTARY DATA IMAGE S1 — Figure S1. [file Image_1.pdf]

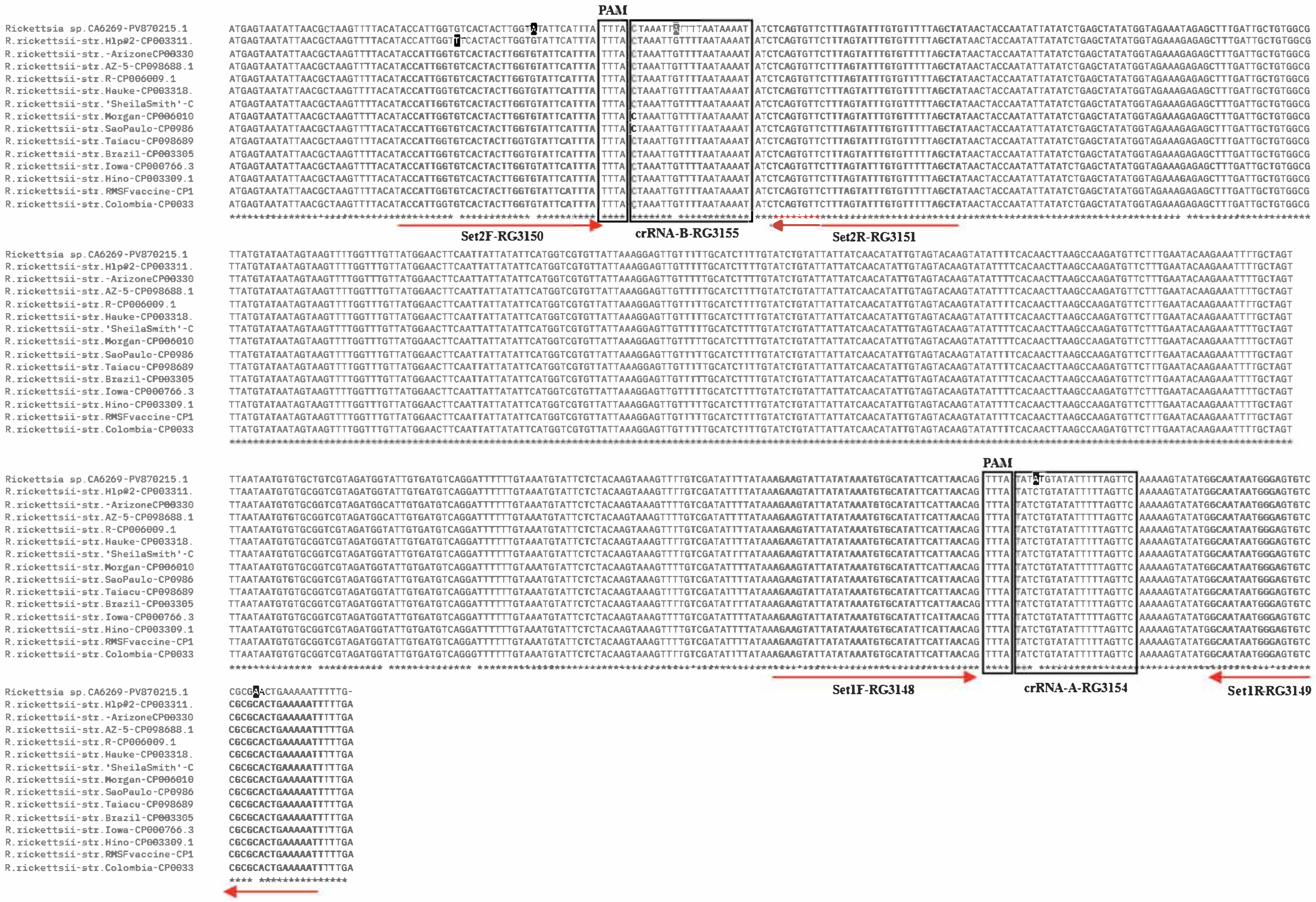

Figure S1. Multiple sequence alignment of the R. rickettsii vut target region used for RPA–LbCas12a assay design. The alignment includes representative R. rickettsii strains and the closest in silico near-neighbor sequence (Rickettsia sp. CA6269). The LbCas12a PAM motif (TTTV) is boxed, and the locations of the two primer sets (Set 1 and Set 2) are indicated with arrows. crRNA spacer positions are labeled (crRNA-A-RG3154 and crRNA-B-RG3155).
